# Endoscopic tympanoplasty with inlay cartilage graft in an university hospital

**DOI:** 10.1016/j.bjorl.2019.10.002

**Published:** 2019-11-16

**Authors:** Thaís de Carvalho Pontes-Madruga, Francisco Bazilio Nogueira Neto, Flávia Alencar de Barros Suzuki, José Ricardo Gurgel Testa, Ektor Tsuneo Onishi

**Affiliations:** a Universidade Federal de São Paulo (UNIFESP), Escola Paulista de Medicina, Otorrinolaringologia, São Paulo, SP, Brazil; b Universidade Federal de São Paulo (UNIFESP), Escola Paulista de Medicina, Fonoaudiologia, São Paulo, SP, Brazil

**Keywords:** Tympanoplasty, Myringoplasty, Tympanic membrane perforation

## Abstract

**Introduction:**

Tympanoplasty is the surgical procedure aimed at the reconstruction of the tympanic membrane and restoration of the sound conducting mechanism. It can be performed with several types of access and grafts and is considered successful when it achieves complete closure of the tympanic perforation and sound conduction improvement.

**Objective:**

To describe the prevalence of successful closure of tympanic perforations and auditory results of endoscopic tympanoplasty with an inlay tragus cartilage graft.

**Methods:**

Retrospective study developed at a tertiary referral hospital. Patients with central tympanic perforations and intact ossicular chains operated with endoscopic tympanoplasty with inlay tragus cartilage graft were included. The neo-tympanum integrity index was evaluated, and the preoperative and postoperative auditory parameters were compared using the paired Student's *t*-test.

**Results:**

We identified 83 endoscopic tympanoplasties with inlay cartilage, of which 63 (76 %) had an intact neo-tympanum and 20 (24 %) had residual perforations. The preoperative air-bone gap was, on average, 18 ± 8.9 dBHL, and the postoperative 11 ± 10 dBHL (*p* = 0.0005), showing reduction in 71 % and complete recovery in 27 %. The mean preoperative speech recognition threshold was 35 ± 13.5 and the postoperative SRT was 27 ± 14.4 (*p* = 0.0002). The preoperative tritonal mean was 34 ± 14.3 and the postoperative was 24 ± 15 (*p* = 0.0002).

**Conclusion:**

In this series, endoscopic tympanoplasties with inlay tragus cartilage graft showed a 76 % prevalence of complete closure of the tympanic perforation, with significant improvement in the auditory parameters.

## Introduction

Tympanoplasty is a procedure aimed at reconstructing the tympanic membrane, restoring round-window protection and the conductive sound mechanism, improving hearing and controlling otorrhea.^1^ Since its introduction in the 1950′s, the surgery has been improved with the addition of several techniques, with retroauricular or transcanal access, grafts such as temporal fascia, fascia lata, veins, perichondrium or cartilage, position of the underlay, overlay or inlay graft in relation to the tympanic membrane, while using a microscope and/or endoscope.^2^, ^3^, ^4^

The technique known as inlay “butterfly” cartilage tympanoplasty was introduced in 1998 by Eavey. It consists in the use of cartilage removed from the tragus, making a graft approximately in the shape of the tympanic perforation, slightly wider and with a groove covering its entire diameter, which should be inserted at the perforation edge, by transcanal access. This technique employs a shorter surgical time and results in better postoperative patient comfort, since it does not involve a tympanomeatal flap or retroauricular incision. Its indication is restricted to tympanic perforations with fully visible margins by transcanal access.^5^, ^6^ The use of the endoscope has helped to popularize this technique by increasing the visibility of perforation edges.

Success in tympanoplasty is defined by the complete closure of the tympanic perforation, with neotympanum formation, absence of lateralization, and good audiological results.^7^ The literature estimates a complete perforation closure rate (anatomical success) ranging from 71%–98% ^8^ and auditory improvement (functional success) of around 60 % for this procedure.^3^, ^9^

## Objective

The aim of this study was to describe the anatomical (intact neotympanum) and functional (gain in auditory parameters) success rate of endoscopic tympanoplasty with inlay tragus cartilage graft in a university hospital.

## Methods

This is a retrospective observational study, developed in a university hospital with a training service for otorhinolaryngology residents.

**Figure 1:**
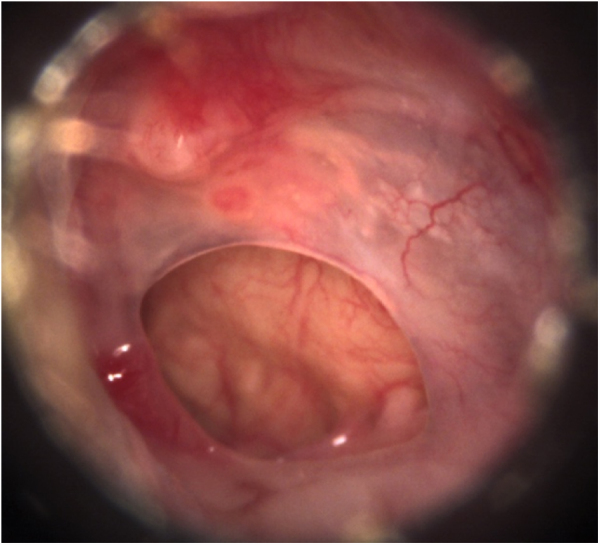
Preoperative otoscopy: central perforations of the tympanic membrane, healthy tympanic cavity mucosa and absence of suppuration.

We included patients with central tympanic perforations, defined as those in which all edges are visible at the otoscopy, occupying a maximum of 25 % of the tympanic membrane area, attributed to chronic non-suppurative otitis media with presumably intact tympanic chains, with absent otorrhea in the three preoperative months, who underwent transcanal endoscopic tympanoplasty with inlay cartilage graft (Fig. 1).

**Figure 2:**
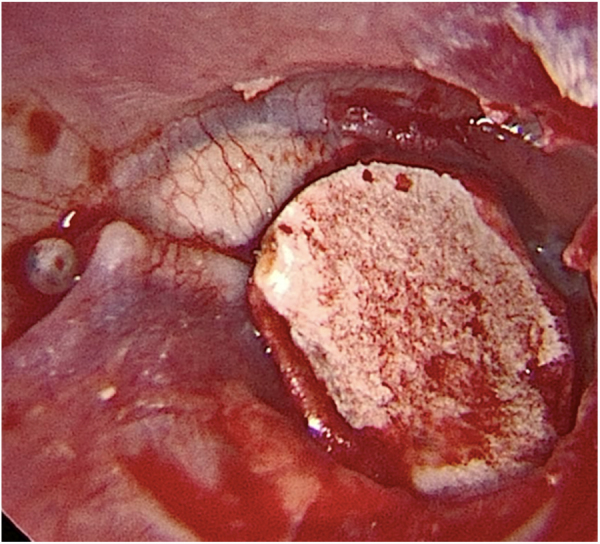
Paper mold adequate for the size and shape of the tympanic perforation.

**Figure 3:**
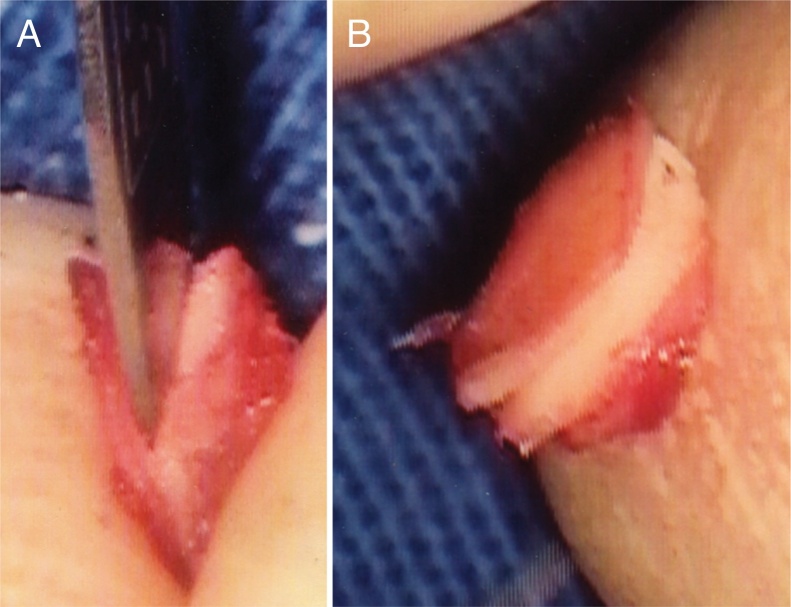
Cartilage graft. A, Making the groove. B, Groove is ready.

**Figure 4:**
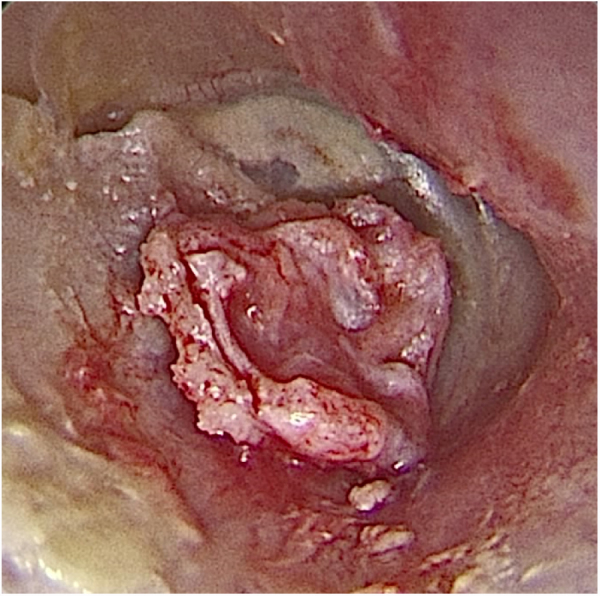
Intraoperative otoendoscopy: cartilage graft with a groove inserted in the perforation and perichondrium facing the external acoustic meatus.

Transcanal endoscopic tympanoplasty with inlay cartilage graft was performed by third-year otorhinolaryngology resident physicians, under the supervision of otologist preceptors, using the following technique: 1) transcanal access with a 0° 4-mm endoscope; 2) scarification of the perforation edges; 3) removal of cartilage fragment from the tragus region; 4) measurement of perforation using Rosen knife (horizontal knife); 5) preparation of paper mold; 6) verification of the shape and dimensions of the perforation with a paper mold; 7) creation of the cartilage graft based on the paper mold, of which dimensions should slightly exceed those of the tympanic perforation, removing the perichondrium from the inner surface (facing the tympanic cavity) and preserving the outer surface (facing the external acoustic meatus); 8) creation of a groove on the entire edge of the cartilage graft, and 9) introduction of the cartilage graft with a groove inserted at the edges of the tympanic perforation (Figure 2, Figure 3, Figure 4).

The exclusion criteria were: age below 12 years; marginal, subtotal or total tympanic perforations; suppurative chronic otitis media; other tympanoplasty techniques that used other types of grafts, another position in relation to the remaining tympanic membrane or access through the microscope, as well as those in which there was ossicular chain manipulation or exploration of the tympanic and mastoid cavity; preoperative otorrhea; absence of essential information registered in medical records.

Following the tympanoplasty, patients are observed for six months, being assessed regarding the integrity of the neotympanum, the presence of otorrhea or other postoperative complications, with audiometry being performed approximately three months after the surgery, which is compared with the preoperative examination. Data were extracted from consultation, audiometry records and surgery descriptions.

According to the norms of Resolution 466/12 of the National Health Council/MOH for Research Involving Humans, this study was submitted to the Research Ethics Committee and was approved under number CAAE 53112816.6.0000.5505.

## Results

Eighty-three endoscopic tympanoplasty procedures were performed with an inlay cartilage graft between 2011 and 2015.

**Figure 5:**
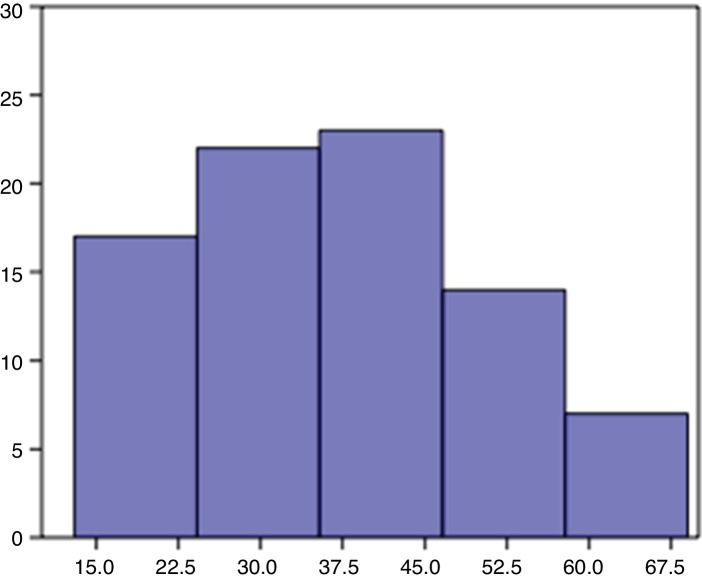
Distribution of patients by age group.

Patient age ranged from 13 to 69 years, with a mean of 37.5 and standard deviation of 13.8. 59 patients were women (71 %) and 24 men (29 %). The procedure laterality was left in 45 (54 %) and right in 38 (46 %) (Fig. 5).

**Figure 6:**
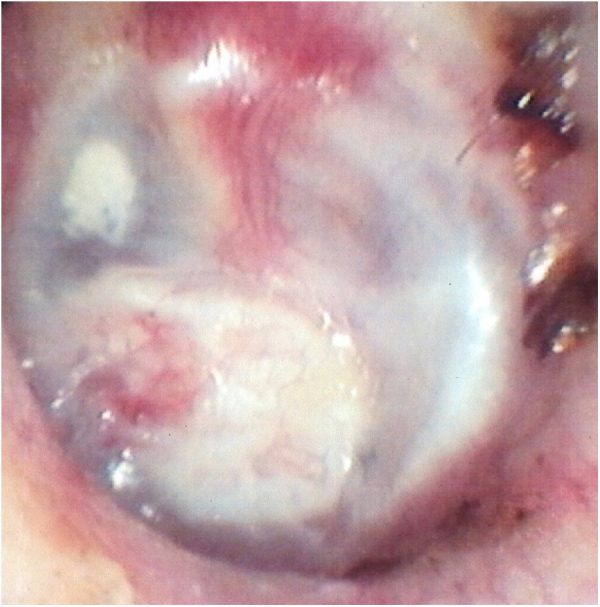
Otoendoscopy in the 6th postoperative month: intact neotympanum after cartilage graft adhesion.

In the 6th postoperative month, 63 patients (76 %) had an intact neotympanum and 20 (24 %) had residual perforations (Fig. 6).

Postoperative otorrhea occurred in 14/83 patients (17 %), affecting 10/20 patients with residual perforation (50 %) and only 4/63 patients with an intact neotympanum (6.3 %), with a statistically significant incidence difference between the groups (*p* < 0.0001). Two patients (2.4 %) were diagnosed with otomycosis during the postoperative follow-up, both with residual perforation.

The mean age of patients with an intact neotympanum was 39 years and 33 years with residual perforation, with no statistical difference (*p* = 0.134). Patients aged up to 20 years had a residual perforation rate of 39 % (5/13), while those older than 20 years had 21 % (15/70) of residual perforation, but the difference was not statistically significant (*p* = 0.287).

The mean preoperative air-bone auditory gap was 18 dBHL and postoperatively 11 dB HL, with a statistically significant difference (*p* = 0.0005). There was a reduction in 59 patients (71 %) and complete recovery in 22 (27 %).

The mean preoperative Speech Recognition Threshold (SRT) was 35 dB, falling postoperatively to 27 dB, a statistically significant difference (*p* = 0.0002), with some reduction in 61 patients (74 %). The preoperative tritonal mean was 34 dB and the postoperative was 24 dB (*p* = 0.0002) (Table 1).

**Table 1:** Evolution of preoperative and postoperative auditory parameters.

Auditory parameters	Preoperative		Postoperative		*p*
	Mean	SD^a^	Mean	SD^a^	
Air-bone auditory gap	17.89	8.88	11.11	9.99	0.0005
Speech Recognition Threshold (SRT)	35.29	13.5	26.89	14.35	0.0002
Tritonal average 500‒1000‒2000 kHz	33.46	14.25	24.44	15.04	0.0002

a SD, Standard deviation.

## Discussion

Transcanal endoscopic tympanoplasty with inlay cartilage graft showed a prevalence of success in establishing an intact tympanic membrane of 76 % (63/83) in this study. For the same endoscopic tympanoplasty technique and a sample with similar age range, Eren described a success rate of 95.5 % (21/22),^10^ Dermihan of 92 % (23/25);^11^ Ulku of 92 % (23/25),^12^ and Karatas of 86.3 % (57/66).^13^ Using this technique in a pediatric population, Karatas described a success rate of 91.4 % (53/58)^14^ and Akyigit in 93.7 % (30/32),^15^ while Isaacson obtained intact tympanic membrane in 55 % of the sample (17/31), microperforations in 32 % (10/31) and larger perforations in 13 % (4/31).^16^ In the present study, the identification of any perforation at the end of the postoperative follow-up was considered as an unsuccessful operation, regardless of the size and its association with the graft.

This series is influenced by the minimal experience of the still-resident surgeons, who have performed the technique for a period shorter than one year. Vartiainen described a frequency of tympanic perforation closure of 78 % of 188 tympanoplasty procedures performed by residents, compared with 95 % of the 594 tympanoplasty procedures performed by preceptors, with two resident-operated patients developing cholesteatomas.^17^ Liu observed a success rate of 81.82 % among 44 tympanoplasty procedures performed by residents, compared with 96.43 % of the 56 surgeries performed by qualified preceptors, with surgeries performed by residents being longer and more costly.^18^ In tympanoplasty performed by residents, Fukuchi describes a success rate of 65 %,^8^ Sirena, of 80 % (24/30),^1^ and Lima, of 95 % (37/39).^19^ In this study and in the aforementioned ones, there were no major complications in residents’ surgeries, but the success rate would justify a more careful supervision, with preceptor intervention whenever necessary.^17^

Endoscopic access has been increasing in otologic surgeries. Modern microscopes provide excellent binocular vision of the operative field and allow a bimanual surgical approach, but incorporate restrictions regarding the visualization of difficult-to-reach recesses. As the light-emitting source is far from the surgical site, the curvature cannot be transposed, making soft tissue retraction and bone removal necessary to visualize certain regions. The endoscope has the advantage of bringing the light source closer to the surgical site, providing a complete and magnified image, with high resolution and capacity to transpose curvatures, making the external acoustic meatus an excellent access route and allowing the visualization of recesses through the use of angled endoscopes. As a major disadvantage, it restricts one of the surgeon’s hands, making it difficult to control bleeding when it occurs. By bringing the light closer to the surgical site, the endoscope can cause thermal injuries. It can be used as an exclusive access or combined with the microscope.^20^, ^21^, ^22^, ^23^, ^24^ In this study, all tympanoplasty procedures were performed using only the endoscope.

Eren advocates the use of the transcanal endoscopic tympanoplasty technique with inlay cartilage to close perforations in the anterior quadrants of the tympanic membrane, of which lower rates of success in tympanoplasty are attributed to vascularization issues,^25^ but in addition mainly to the difficulty of complete visualization with the microscope, due to the anterior curvature of the external acoustic meatus, often requiring retroauricular access or canaloplasty. The use of the endoscope facilitates the visualization of the anterior border, transposing the curvature of the meatus, just as the inlay positioning of the graft would avoid the distance between a possible posterior tympanomeatal flap and the anterior perforation.^10^

Techniques with placement of the underlay or overlay graft in relation to the tympanic membrane involve flap incisions in the external acoustic meatus, which may granulate, cause pain, as well as ear fullness and temporary hearing loss related to the filling of the outer and middle ears with bandages. The transcanal inlay technique eliminates retroauricular or meatal incisions, reduces surgical time and the need to bandage the ears, and minimizing hearing problems in the immediate postoperative period. The cartilage graft donor site, the tragus, has low morbidity and shows better cosmetic results.^14^, ^16^ In the literature, the success rates of inlay cartilage tympanoplasty do not differ from the underlay techniques.^3^, ^9^, ^26^

In this study, there was a significant improvement in audiometric parameters. The gap was reduced in 71 % of patients, with closure in 27 % and average gain of 7 dB. Other studies with endoscopic transcanal tympanoplasty with inlay cartilage reported an average gap gain ranging from 8 to 12 dB.^10^, ^12^, ^13^, ^14^, ^15^ The strength and stiffness of the cartilage provide greater graft stability, but there is concern that these grafts characteristics have a negative effect on sound conduction.^27^, ^28^

In our experience with surgical training of otolaryngology residents, the view provided by the endoscope facilitates anatomical guidance in otologic surgeries. Among the tympanoplasty techniques performed on our service, the insertion of inlay cartilage via transcanal route has the shortest learning curve and is a good opportunity to train residents on the use of the endoscope, showing less bleeding and less bimanual demand than the underlay technique, of which detachment from the tympanomeatal flap involves bleeding, requiring skill for its performance with one hand. Its indication is restricted to perforations with all edges present, allowing the complete insertion of the cartilage groove. For marginal perforations, the underlay technique with temporal fascia graft is preferred.

## Conclusion

The success rate (intact neotympanum) of transcanal endoscopic tympanoplasty with inlay cartilage graft was 76 %. There was a 71 % reduction in the air-bone auditory gap, with a complete recovery of 27 % and an average gain of 7 dB.

## Conflicts of interest

The authors declare no conflicts of interest.
